# Supplementary material for: Evaluating the predictive strength of the LACE index in identifying patients at high risk of hospital readmission following an inpatient episode: a retrospective cohort study
Source: BMJ Open. 2017 Jul 13;7(7):e016921. doi: 10.1136/bmjopen-2017-016921 (PMC5726103; doi:10.1136/bmjopen-2017-016921)
Supplement: Author's manuscript [file bmjopen-2017-016921.draft_revisions.pdf]

BMJ Open

Evaluating the predictive strength of the LACE index in identifying patients at high risk of hospital readmission following an inpatient episode: retrospective cohort study

For peer review only

Journal:	*BMJ Open*
Manuscript ID	bmjopen-2017-016921
Article Type:	Research
Date Submitted by the Author:	20-Mar-2017

Complete List of Authors:	Damery, Sarah; University of Birmingham, Primary Care Clinical Sciences<br>Combes, Gill; University of Birmingham, Institute of Applied Health Research

**Primary Subject Heading**:	Health policy
Secondary Subject Heading:	Evidence based practice
Keywords:	LACE, readmissions, case finding, risk stratification, hospital

SCHOLARONE™
Manuscripts

Evaluating the predictive strength of the LACE index in identifying patients at high risk of hospital readmission following an inpatient episode: retrospective cohort study

Dr Sarah Damery, Research Fellow, Institute of Applied Health Research, College of Medical and Dental Sciences, University of Birmingham, Edgbaston, West Midlands, UK, B15 2TT

Dr Gill Combes, CLAHRC West Midlands Research Lead for Chronic Diseases Theme, Institute of Applied Health Research, College of Medical and Dental Sciences, University of Birmingham, Edgbaston, West Midlands, UK, B15 2TT

Corresponding author:
Dr Sarah Damery
Research Fellow
Institute of Applied Health Research
College of Medical and Dental Sciences
University of Birmingham
Edgbaston
West Midlands
UK
B15 2TT

Email: s.l.damery@bham.ac.uk

For peer review only

For peer review only - http://bmjopen.bmj.com/site/about/guidelines.xhtml

Abstract

Objective:
To assess how well the LACE index and its constituent elements predicts 30-day hospital readmission and to determine whether other combinations of clinical or sociodemographic variables may enhance prognostic capability.

Design:
Retrospective cohort study with split sample design for model validation.

Setting:
One large hospital Trust in the West Midlands.

Participants:
All alive-discharge adult inpatient episodes between 1st January 2013 and 31st December 2014.

Data sources:
Anonymised data for each inpatient episode were obtained from the hospital information system. These included age at index admission, gender, ethnicity, admission and discharge date, length of stay, treatment specialty, admission type, admission source, discharge destination. Data were also obtained on comorbidities, number of accident and emergency (A&E) visits in the six months before the index admission, and whether a patient was readmitted within 30 days of index discharge.

Outcome measures:
Clinical and patient characteristics of readmission vs. non-readmission episodes, proportion of readmission episodes at each LACE score from 0 to 19, regression modelling of variables associated with readmission to assess the effectiveness of LACE and other variable combinations to predict 30-day readmission.

Results:
Increasing LACE score and each of its individual components were independent predictors of readmission (AUC 0.773; 95% CI: 0.768 to 0.779 for LACE; AUC 0.806; 95% CI: 0.801 to 0.812 for the four LACE components). A LACE score of 11 was most effective at distinguishing between higher and lower risk patients. However, only 25% of readmission episodes occurred in the higher scoring group. A model combining A&E visits and hospital episodes per patient in the previous year was more effective at predicting readmission (AUC 0.815; 95% CI: 0.810 to 0.819).

Conclusions:
Although LACE shows good discriminatory power in statistical terms, it may have little added value over and above clinical judgement in predicting a patient's risk of hospital readmission.

Word count:
2872

Keywords: LACE, readmissions, case finding, risk stratification, hospital

For peer review only - http://bmjopen.bmj.com/site/about/guidelines.xhtml

STRENGTHS AND LIMITATIONS OF THIS STUDY

- This study assessed the characteristics associated with 30-day hospital readmission in a large hospital Trust in the West Midlands
- A split sample design allowed mode development and statistical testing to be undertaken in one half of the dataset and the results validated in a representative sample of inpatient episodes from a directly comparable population
- In focusing on a general medical population, the study evaluated the LACE index in a context similar to that in which it was originally developed
- Readmission rates may have been underestimated as we were unable to identify cases where a patient may have been readmitted to another hospital

For peer review only

For peer review only - http://bmjopen.bmj.com/site/about/guidelines.xhtml

INTRODUCTION

In recent years, developing effective ways to reduce rates of patient readmission following an episode of acute care has become a key health policy focus in many developed economies.[1] In 2011/12, the average 30-day readmission rate in England was 5.6%, with variation across acute Trusts of between 3 and 10%.[2] It is estimated that 30-day readmissions incur annual costs in excess of £2.5 billion for the National Health Service (NHS),[3] and in 2011, the Department of Health introduced a policy of non-payment to hospitals in England for emergency readmissions within 30 days of discharge following an elective admission. In 2012, this was extended to encompass both elective and emergency admissions. The policy applies to all clinical areas except those where it is considered inappropriate to withhold payment (e.g. readmission in cancer or dialysis patients), and operates by establishing local readmission thresholds following clinical review of readmissions at a given Trust and determining the proportion that could be considered avoidable.[4] In a financially straitened NHS, the prospect of incurring financial penalties for 30-day readmissions has created a strong incentive for hospital Trusts to reduce readmission rates.

The most common clinical reasons for readmission are infections, and complications related to medical care or long term conditions,[5] and it is thought that readmission rates can be reduced substantially if at-risk patients can be identified before discharge and offered supportive interventions as inpatients or after discharge. However, interventions are most likely to be effective if they are targeted towards patients at highest risk of future hospital use,[6] as being able to distinguish between patients who will not require readmission and those who are likely to be readmitted has implications for the cost-effectiveness of readmission avoidance interventions.[7-9] Identifying at-risk patients effectively relies on accurate case finding,[10] and a large number of predictive models have been used both within the NHS,[3,11-13] and internationally,[14-16] to varying degrees of success.[17-19] Predictive models differ in the type and scope of data items they include and the time period over which they seek to predict readmission risk.[20] When choosing an appropriate model, a trade-off often needs to be made between complexity - the number of data items required - and practicality of application in clinical practice.[21]

One widely used predictive tool is the LACE index,[22] which uses routinely collected clinical and administrative data to generate a risk score of between 0 and 19 for individual patients, where higher scores indicate an increased risk of readmission. Scores are based on four features of an inpatient hospital episode: length of stay, admission type, comorbidities and the number of Accident and Emergency (A&E) visits made by a patient in the six months prior to their initial admission. Scores over a specific threshold can be used to 'flag' at-risk patients for whom interventions may be appropriate. Although widely used, the evidence base for LACE is uncertain. Some studies have found it to be an effective predictor of readmission,[23,24] whereas others have demonstrated poor prognostic ability, particularly when applied to specific patient sub-groups.[25,26] The literature on case finding tools emphasises the importance of local validation before implementation, since each hospital has a patient case mix that reflects their surrounding population and may require a locally-calibrated score threshold.[13] A modified version of the LACE index has been developed,[27] that gives greater weight to patient comorbidities, which are considered a key driver of readmissions.[21] This study analysed data from a large hospital Trust in the West Midlands to assess how well the (modified) LACE index and each of its constituent elements predicts 30-day readmission and to determine whether a model based on other combinations of clinical or patient variables may enhance prognostic capability.

METHODS

Sampling

The study used a retrospective cohort design with a split sample to allow the findings to be internally validated. All alive-discharge adult inpatient episodes at a large hospital Trust in the West Midlands over a

For peer review only - http://bmjopen.bmj.com/site/about/guidelines.xhtml

two year period (1st January 2013 to 31st December 2014) were included. Anonymised sociodemographic and clinical data were obtained for each episode (termed the 'index admission') from the hospital information system. Sociodemographic data included patient age at index admission, gender and ethnic group. Clinical data relating to the index admission included: date of admission and discharge, length of stay (LoS), ICD10 code, primary diagnosis, treatment specialty, Health Research Group (HRG) code, admission type (emergency, elective, day case), admission source and discharge destination (e.g. usual place of residence, other NHS institution). Data were also obtained on patient comorbidities, number of A&E visits in the six months before the index admission, whether the index episode was followed by readmission within 30 days of the index discharge date, and if so, the date of readmission and treatment specialty.

Data analysis

A LACE score was calculated based on LoS (0 to 6 points), admission type (0 to 3 points), comorbidity (0 to 6 points) and previous A&E attendance (0 to 4 points), giving a total score between 0 and 19 for each inpatient episode (Table 1).

Table 1: Components of the LACE index and values assigned for each

Attribute	Value	Points
Length of stay	Less than 1 day	0
	1 day	1
	2 days	2
	3 days	3
	4 to 6 days	4
	7 to 13 days	5
	14 or more days	6
Acute admission	Inpatient	3
	Observation	0
Comorbidity (scores cumulative to a maximum of 6)	No prior history	0
	Diabetes without complications, cerebrovascular disease, history of myocardial infarction (MI), peripheral vascular disease (PVD), peptic ulcer disease (PUD)	1
	Mild liver disease, diabetes with end organ damage, congestive heart failure (CHF), chronic obstructive pulmonary disease (COPD), cancer, leukaemia, lymphoma, any tumour, cancer, moderate to severe renal disease	2
	Dementia or connective tissue disease	3
	Moderate/severe liver disease or human immunodeficiency virus (HIV) infection	4
	Metastatic cancer	6
A&E visits during previous 6 months	0 visits	0
	1 visits	1
	2 visits	2
	3 visits	3
	4 or more visits	4

For peer review only

For peer review only - http://bmjopen.bmj.com/site/about/guidelines.xhtml

Any patient episodes with missing data were removed from the dataset. The dataset was then split in half at random to create a derivation cohort for statistical testing and a separate cohort for validation of the findings. All continuous variables were summarised using medians and interquartile ranges (IQR). Univariate comparisons of these variables across the readmitted/non-readmitted groups used the non-parametric Mann-Whitney *U*-test, and X^2 tests compared the characteristics of readmitted vs. non-readmitted patients for variables with categorical data. Univariate odds ratios (OR) and their 95% confidence intervals (CI) were calculated for sociodemographic and clinical variables and for each component of the LACE index to test the association between variable subgroups and readmission. Finally, binary logistic regression modelling using the enter method was used to test the strength of different combinations of variables in predicting the likelihood of 30 day readmission. Model strength was described using OR, and the area under the receiver operating characteristic (ROC) curve described using the *c*-statistic. The findings for each model were then validated using the patient episodes in the validation cohort. All statistical analyses were undertaken using SPSS (version 21, SPSS Inc., Chicago IL).

RESULTS

Sample characteristics

The full dataset included 183843 patient episodes (103493 individual patients). After splitting the dataset to create the derivation and validation cohorts, subsequent analyses were performed on the derivation cohort prior to validation, which contained data on 91,922 separate admission episodes (representing 51747 individual patients) (Table 2).

Table 2: Characteristics of readmission vs. non-readmission episodes

Variable	Grouping	Total episodes (%)	Readmitted (%)	Not readmitted (%)	Comparison*
Patient age	Median, range (IQR)	55.0, 18 to 106 (37 to 72)	64.0, 18 to 105 (44 to 78)	55.0, 18 to 106 (37 to 71)	p<0.0001
Gender	Male	39001 (42.4)	3545 (9.1)	35456 (90.9)	X^2=175.1; p<0.0001
	Female	52921 (57.6)	3562 (6.7)	49359 (93.3)	
Index LoS (days)	Median, range (IQR)	0.0, 0 to 301 (0.0 to 2.0)	1.0, 0 to 223 (0.0 to 5.0)	0.0, 0 to 301 (0.0 to 1.0)	p<0.0001
Admission type	Emergency	46922 (51.0)	6005 (12.8)	40917 (87.2)	X^2=3573.4; p<0.0001
	Elective	7243 (7.9)	410 (5.7)	6833 (94.3)	
	Day case	37757 (41.1)	692 (1.8)	37065 (98.2)	
Comorbidity score**	0	74274 (80.8)	5083 (6.8)	69191 (93.2)	X^2=1126.9; p<0.0001
	1	14984 (16.3)	1820 (12.1)	13164 (87.9)	
	2	2147 (2.3)	29 (1.4)	2118 (98.6)	
	3	514 (0.6)	172 (33.5)	342 (66.5)	
	4	3 (0.0)	3 (100.0)	0 (0.0)	
	6	0 (0.0	0 (0.0)	0 (0.0)	
A&E visits in previous 6 months	Median, range (IQR)	1.0, 1 to 121 (1.0 to 2.0)	2.0, 1 to 107 (1.0 to 4.0)	1.0, 1 to 121 (1.0 to 2.0)	p<0.0001

* Continuous variables were compared using the Mann-Whitney *U*-test and categorical variables were compared using the X^2 test. ** Comorbidity score does not relate to number of comorbidities; scores are assigned based on severity of comorbidities

Median patient age in the derivation cohort was 55 (IQR: 37 to 72), and male patients accounted for 42.4% of hospital episodes (n=39001). The median LoS of the index admission was 0 days (IQR: 0 to 2). 51.0% of

For peer review only - http://bmjopen.bmj.com/site/about/guidelines.xhtml

episodes followed emergency admission (n=46922). The majority of patients had a comorbidity score of 0 (80.8%) and the median number of A&E visits in the 6 months prior to the index episode was 1 (IQR: 0 to 1).

Characteristics of readmitted vs. non-readmitted patients

A total of 7107 inpatient episodes were followed by a readmission within 30 days (7.7% readmission rate, 4541 individual patients). 1218 patients (2.4%) accounted for 53.1% of all readmission episodes. A comparison of the characteristics of episodes that resulted in readmission vs. those that did not showed statistically significant differences for all variables. Readmitted patients were significantly more likely to be older than those who were not readmitted (median age 64 vs. 55), men had significantly higher readmission rates than women (9.1% vs. 6.7%), and emergency admissions were significantly more likely to result in readmission than elective or day case admissions (12.8% vs. 5.7% and 1.8% respectively). Median LoS in readmitted patients was 1 day (IQR: 0 to 5); median A&E visits in the previous 6 months was 2 (IQR: 1 to 4), and higher comorbidity scores were significantly associated with readmission, with 33.5% of patients with a comorbidity score of 3 being readmitted within 30 days.

Readmission episodes and LACE score

The median LACE score in the derivation cohort was 5 (range 3 to 17, IQR: 3 to 7) (Table 3). The median score for readmission episodes was significantly higher than the median score for episodes that did not result in readmission (8.0 with IQR 6 to 11 vs. 5.0 with IQR 3 to 7; $p<0.0001$). Readmission rates more than doubled between a LACE score of 10 and 11, suggesting that 11 may be the optimum threshold for distinguishing between patients at a lower or higher risk of readmission. However, the proportion of total readmissions represented by LACE scores 11 and above was only 25.3% of the total (1795/7107), thus nearly three quarters of readmissions occurred in patients scoring lower than the cut-off point.

Table 3: Proportion of readmission episodes at each LACE score

LACE score*	Total episodes (%)	Readmitted (%)	Not readmitted (%)
3	26478 (28.8)	302 (1.1)	26176 (98.9)
4	13798 (15.0)	561 (4.1)	13237 (95.9)
5	14152 (15.4)	676 (4.8)	13476 (95.2)
6	10656 (11.6)	753 (7.1)	9903 (92.9)
7	8637 (9.4)	1045 (12.1)	7592 (87.9)
8	5800 (6.3)	913 (15.7)	4887 (84.3)
9	4136 (4.5)	551 (13.3)	3585 (86.7)
10	3242 (3.5)	511 (15.8)	2731 (84.2)
11	2379 (2.6)	815 (34.3)	1564 (65.7)
12	1570 (1.7)	633 (40.3)	937 (59.7)
13	751 (0.8)	231 (30.8)	520 (69.2)
14	217 (0.2)	81 (37.3)	136 (62.7)
15	104 (0.1)	35 (33.7)	69 (66.3)
16	1 (0.0)	0 (0.0)	1 (100.0)
17	1 (0.0)	0 (0.0)	1 (100.0)

* Percentages for readmitted and not readmitted are calculated according to variable grouping e.g. % of episodes scoring 3 on the LACE index which resulted in readmission vs. those that did not

For peer review only

For peer review only - http://bmjopen.bmj.com/site/about/guidelines.xhtml

Univariate logistic regression

Univariate binary logistic regression assessed the association between individual variables and the likelihood of readmission (Table 4). All variables were statistically significant to the p<0.0001 level. For each unit increase in patient age, the likelihood of readmission rose by 1.7%. Females were significantly less likely to be readmitted than males, despite constituting a larger proportion of index admissions (OR 0.72; 95% CI: 0.69 to 0.76). Increasing LACE score was significantly associated with 30 day readmission, with each point increase in score associated with a 42% increase in the likelihood of readmission. The variable with the strongest association with readmission was emergency admission – index episodes which were a result of emergency admission were nearly 8 times more likely to be followed by readmission than those in the reference group of day case surgery (OR 7.87; 95% CI: 7.26 to 8.52).

Table 4: Univariate logistic regression of variables potentially associated with readmission

Variable	Grouping	P value	Odds ratio (95% CI)
Patient age	Continuous	<0.0001	1.03 (1.02 to 1.04)
Patient gender	Male	Reference	Reference
	Female	<0.0001	0.72 (0.69 to 0.76)
Length of stay	Continuous	<0.0001	1.04 (1.03 to 1.04)
Admission type	Day case	Reference	Reference
	Elective	<0.0001	3.21 (2.84 to 3.64)
	Emergency	<0.0001	7.87 (7.26 to 8.52)
Comorbidity score	Continuous	<0.0001	1.28 (1.25 to 1.31)
A&E visits in previous 6 months	Continuous	<0.0001	1.39 (1.38 to 1.41)
LACE score	Continuous	<0.0001	1.42 (1.41 to 1.43)
Episodes per patient in previous year	Continuous	<0.0001	1.06 (1.05 to 1.06)

Multivariate logistic regression

Four multivariate logistic regression models were constructed to assess different potential predictors of 30 day readmission (Table 5). The first included LACE index score only. LACE score was highly significant as a predictor of readmission (p<0.0001), with an AUC of 0.773 (95% CI: 0.768 to 0.779) and R^2 of 0.180. This is higher than the *c*-statistic for LACE score as a predictor of readmission found by van Walraven et al who developed the LACE index. However, the other three models all had a higher *c*-statistic: model 2, which included all eight variables from the univariate analysis had a *c*-statistic of 0.820 (95% CI: 0.815 to 0.825) and R^2 of 0.240. In this model, whilst all included variables were significant at the p<0.0001 level, the direction of effect for some variables differed from the univariate testing. Both increasing length of stay and higher comorbidity score were associated with a *lower* likelihood of readmission in this model when the effects of other variables was controlled for. Model 3 included the four components of the LACE index, and was a better predictor of readmission than LACE score alone, with a *c*-statistic of 0.806 (0.801 to 0.812). In the fourth model, which included only A&E visits and number of episodes per patient as variables, was a better model for predicting readmission than models 1 and 3 and had only a marginally lower *c*-statistic than model 2 which was the most complex in terms of number of variables included (AUC=0.815; 95% CI: 0.810 to 0.819).

For peer review only - http://bmjopen.bmj.com/site/about/guidelines.xhtml

Model validation

The models developed using the derivation cohort were tested for validity in the validation cohort. The validation cohort did not differ from the derivation cohort in any of the sociodemographic of clinical variables assessed. It included 91921 episodes of care, of which 7008 (7.6%) were followed by a readmission within 30 days. The *c*-statistic of the logistic regression models developed from the derivation cohort were 0.767 (0.761 to 0.772) for model 1, 0.814 (0.809 to 0.189) for model 2, 0.800 (0.794 to 0.805) for model 3 and 0.812 (0.807 to 0.817) for model 4.

Table 5: Binary logistic regression models assessing the probability of readmission

Variable	P value	Odds ratio (95% CI)	AUC (95% CI); R^2
Model 1: LACE score only			
LACE score	<0.0001	1.42 (1.41 to 1.43)	AUC=0.773 (0.768 to 0.779); R^2=0.180
Model 2: All variables from univariate analysis			
Age	<0.0001	1.01 (1.01 to 1.02)	AUC=0.820 (0.815 to 0.825); R^2=0.240
Gender (female)	<0.0001	0.90 (0.85 to 0.95)	
Length of stay	<0.0001	0.98 (0.98 to 0.99)	
Admission type (emergency)	<0.0001	4.18 (3.77 to 4.64)	
Comorbidity score	<0.0001	0.96 (0.94 to 0.98)	
A&E visits in previous 6 months	<0.0001	1.12 (1.11 to 1.13)	
LACE score	<0.0001	1.23 (1.21 to 1.25)	
Episodes per patient	<0.0001	1.07 (1.06 to 1.07)	
Model 3: Four components of the LACE index			
Length of stay	<0.0001	1.02 (1.01 to 1.03)	AUC=0.806 (0.801 to 0.812); R^2=0.193
Admission type (emergency)	<0.0001	4.79 (4.40 to 5.22)	
Comorbidity score	<0.0001	1.30 (1.26 to 1.33)	
A&E visits in previous 6 months	<0.0001	1.28 (1.27 to 1.29)	
Model 4: Reduced complexity model			
A&E visits in previous 6 months	<0.0001	1.36 (1.35 to 1.38)	AUC=0.815 (0.810 to 0.819); R^2=0.130
Episodes per patient in previous year	<0.0001	1.03 (1.02 to 1.04)	

DISCUSSION

The primary aim of this study was to assess how well the modified LACE index was able to predict 30-day readmission in a cohort of patients admitted to a large secondary care Trust over a two year period. Increasing LACE score and the four individual components comprising the LACE index were all independent predictors of readmission. The proportion of admissions episodes resulting in readmission increased substantially at a LACE score cut-off of 11, which would suggest that this is an appropriate threshold to use when deciding whether to provide enhanced inpatient and/or post-discharge care to prevent unplanned readmission. However, although a large proportion of admissions episodes that scored 11+ on the LACE index were followed by a readmission within 30 days, this corresponded to comparatively few absolute numbers of patients. Only 25% of all readmissions occurred in the higher scoring group, whilst the remaining 75% occurred following episodes of care that scored <11 on the index. This differs from other studies that have assessed the effectiveness of different risk thresholds for LACE, which typically saw a higher proportion of all readmissions occurring in the patient group that scored above the chosen threshold.[23,28]

For peer review only - http://bmjopen.bmj.com/site/about/guidelines.xhtml

Whilst implementing a lower LACE score threshold would improve the likelihood of identifying at-risk patients, a large number of these patients would not go on to be readmitted. In a health service facing substantial resource constraints, the LACE tool is unlikely to have the sensitivity and specificity that would make it a useful addition to clinical practice.

A number of studies have assessed the performance on the LACE index in predicting unplanned readmissions but these have typically been conducted in small patient populations,[29,30] or in specific patient groups such as cardiovascular disease,[25,29,31] COPD,[30] or older people.[26] The patient cohort included in this study was large, and the analysis had good statistical power to detect differences between groups. In focusing on a general medical population, our study evaluated the LACE index in a context similar to that in which it was originally developed in terms of patient characteristics and incidence of comorbidity.[22,27] The split sample design allowed model development and statistical testing to be carried out in one half of the dataset, and the results validated in a representative sample of inpatient episodes from a directly comparable population. Readmission rates may have been underestimated in the hospital data used for this study, as we were unable to identify instances where a discharged patient may have subsequently been readmitted to another hospital.

Multiple factors typically contribute to readmission rates, and there are limits on the extent to which unplanned readmissions can be avoided.[32,33] High readmission rates are often thought to indicate sub-optimal patient management, but they are most likely to be driven by difficulties in managing patient transitions to other health and social care settings, a lack of community resources for patient follow-up, or influenced by the patient's home environment.[34] A retrospective analysis of 82 million routinely collected hospital records in England between 2004-2010 found that only 30% of unplanned readmissions were deemed avoidable.[35] Therefore, lowering readmission rates for patients with chronic or relapsing conditions, or patients readmitted with a different diagnosis from their index admission, poses a significant challenge. Conversely, avoiding readmissions in patients presenting with a recurrence or continuation of the issue that led to their initial hospitalisation, or for those who are readmitted with an avoidable complication related to their index admission should be a priority for hospital Trusts, which is a key reason that case finding tools are increasingly being tested in the hospital setting. Although a number of increasingly complex tools have been developed in recent years, such as PARR-30,[36] LACE+,[37] and HOSPITAL,[38] the intuitive appeal of LACE lies in its simplicity and use of routinely collected hospital data.

This study suggests that despite a number of sociodemographic and clinical variables being strongly associated with hospital readmission in statistical terms, the added value of the LACE tool over and above clinical judgement remains equivocal. However, the fact that small gains in model accuracy and discriminatory power can be made by testing different combinations of potential predictor variables derived from routinely collected hospital administrative data may indicate that the accuracy of case finding could be improved through the addition of locally-relevant clinical or sociodemographic factors.[39,40] In this study, the predictive model with the least discriminatory power was based on LACE score alone. Model 2, which included eight predictor variables, was only marginally better at predicting readmission than model 4 which included only two variables: A&E visits and the number of admissions per patient in the previous 12 months. This would suggest – in a cohort of general medical admissions - that a simpler model could outperform the more complex LACE tool in accurately identifying patients at risk of readmission. Our analysis showed that 2.4% of patients in the cohort accounted for 53.1% of all readmission episodes. Being able to identify the small group of patients who use a disproportionate amount of healthcare resources is the first step towards developing solutions to prevent repeat hospitalisations in this population.[41] Future research should focus on the development of locally-tailored screening tools to identify these patients.

For peer review only - http://bmjopen.bmj.com/site/about/guidelines.xhtml

CONCLUSION

Although LACE shows good discriminatory power in statistical terms, it may have little added value over and above clinical judgement in predicting a patient's risk of hospital readmission. Nevertheless, if used as a screening tool alongside clinical judgement, a locally-tailored risk score based on specific clinical or sociodemographic variables relevant to the inpatient population admitted to a particular hospital Trust may increase case finding accuracy. This could allow clinicians to effectively discriminate between patients who are likely to have an unplanned admission within 30 days of discharge and those that will not.

For peer review only

DECLARATIONS

Declaration of competing interests: All authors have completed the Unified Competing interest form at www.icmje.org/coi_disclosure.pdf (available on request from the corresponding author) and declare no support from any organisations for the submitted work; no financial relationships with any organisations that might have an interest in the submitted work in the previous three years; no other relationships or activities that could appear to have influenced the submitted work.

Acknowledgements: We would like to thank Martin Chadderton for providing the data from the hospital information system, and Roger Steadman for clinical guidance on the study.

Details of contributors: SD and GC designed the study. SD wrote the study protocol. SD undertook data analysis, with input from GC as needed. SD drafted and revised the paper and is guarantor for the work. GC critically revised the paper for intellectual content. All authors gave final approval of the manuscript and are accountable for all aspects of the accuracy and integrity of the work.

Ethics approval: Ethical approval was obtained from the University of Birmingham Research Ethics Committee (Ref: ERN_14-0914). Research governance approval was obtained from the R&D office of Sandwell and West Birmingham Hospitals NHS Trust (Ref: 14MISC40).

Funding: This research was funded by the National Institute of Health Research (NIHR) Collaboration for Leadership in Applied Health Research and Care West Midlands (CLAHRCWM).

Role of study sponsor and funder: The study sponsor and funder had no role in the study design, in the collection, analysis and interpretation of data, in the writing of the report, and in the decision to submit the article for publication.

Independence from funders: This paper presents independent research funded by the National Institute of Health Research (NIHR) Collaboration for Leadership in Applied Health Research and Care West Midlands (CLAHRCWM). The views expressed are those of the authors and not necessarily those of the NHS, the NIHR or the Department of Health.

Data access: All authors had full access to all of the data (including statistical reports and tables) in the study and can take responsibility for the integrity of the data and the accuracy of the data analysis.

Transparency: The guarantor (SD) affirms that the manuscript is an honest, accurate and transparent account of the study being reported; no important aspects of the study have been omitted, and any discrepancies from the study as planned and registered have been explained.

Data sharing: No additional data available.

For peer review only

For peer review only - http://bmjopen.bmj.com/site/about/guidelines.xhtml

REFERENCES

1. Conroy S, Dowsing T. What should we do about hospital readmissions? Age Ageing 2012;41(6):702-4.

2. SG2. Reducing 30-day readmissions. SG2 Service kit; June 2011. https://www.hsj.co.uk/Journals/2/Files/2011/6/15/Sg2_Service%20Kit_Reducing%2030-Day%20Readmissions.pdf. Accessed 15th March 2014.

3. Billings J, Blunt I, Steventon A, Gheorghiou T, Lewis G, Bardsley M. Development of a predictive model to identify inpatients at risk of re-admission within 30 days of discharge (PARR-30). BMJ Open 2012;00:e001667.

4. Monitor and NHS England. 2016/17 National Tariff Payment System. Monitor, 2016; London.

5. Robinson P. Hospital readmissions and the 30 day threshold. CHKS Marketing Intelligence Report, 2016; London.

6. Ross S, Curry N, Goodwin N. Case management: what it is and how it can best be implemented. The Kings Fund, London: 2011.

7. Purdy S. Avoiding hospital admissions. What does the research evidence say? The King's Fund, London; 2010.

8. Dixon J, Bardsley M. Predictive risk modelling using routine data: underexploited potential to benefit patients. Journal of Health Services Research Policy 2012;17(3):131-2.

9. Billings J, Gheorghiou T, Blunt I, Bardsley M. Choosing a model to predict hospital admission: an observational study of new variants of predictive models for case finding. BMJ Open 2013;3:e003352.

10. Lewis G, Curry N, Bardsley M. How predictive modelling can help reduce risk and hospital admissions. Nuffield Trust, London: 2011.

11. Donnan PT, Dorward DWT, Mutch B, Morris AD. Development and validation of a model for predicting emergency admissions over the next year (PEONY). Arch Intern Med 2008;168(13):1416-22.

12. Chenore T, Pereira Gray DJ, Forrer J, Wright C, Evans PH. Emergency hospital admissions for the elderly: insights from the Devon Predictive Model. Journal of Public Health 2013;35(4):616-23.

13. Gheorghiou T, Billings J, Bardsley M. New predictive case finding models for the NHS. Nuffield Trust, London: 2013.

14. Halfon P, Eggli Y, Pretre-Rohrbach I, Meylan D, Marazzi A, Burnand B. Validation of the potentially avoidable hospital readmission rate as a routine indicator of the quality of hospital care. Med Care 2006;44(11):972-81.

15. Silverstein MD, Qin H, Mercer SQ, Fong J, Haydar Z. Risk factors for 30-day hospital readmission in patients >65 years of age. Proc (Bayl Univ Med Cent) 2008;21(4):363-72.

16. Howell S, Coory M, Martin J, Duckett S. Using routine inpatient data to identify patients at risk of hospital admission. BMC Health Serv Res 2009;9:96.

For peer review only

17. Hansen LO, Young RS, Hinami K, Leung A, Williams MV. Interventions to reduce 30-day hospitalization: a systematic review. Ann Intern Med 2011;155:520-28.

18. Kansagara D, Englander H, Salanitro A, Kagen D, Theobald C, Freeman M, Kripalani S. Risk prediction models for hospital readmission: a systematic review. JAMA 2011;306(15):1688-98.

19. Zhou H, Della PR, Roberts P, Goh L, Dhaliwal SS. Utility of models to predict 28-day or 30-day unplanned hospital readmissions: an updated systematic review. BMJ Open 2016;6:e011060.

20. Department of Health. Long term conditions compendium of information. Department of Health, London; 2012.

21. Williams S, Bottle A, Aylin P. Length of hospital stay and subsequent emergency readmission. BMJ 2005; 331:371.

22. Van Walraven C, Dhalla IA, Bell C, Etchells E, Stiell IG, Zarnke K, Austin PC, Forster AJ. Derivation and validation of an index to predict early death or unplanned readmission after discharge from hospital to the community. CMAJ 2010;182(6):551-7.

23. Gruneir A, Dhalla IA, van Walraven C, Fisher HD, Camacho X, Rochon PA, Anderson GM. Unplanned readmissions after hospital discharge among patients identified as being at high risk for readmission using a validated predictive algorithm. Open Med 2013;5:e104-11.

24. Spiva L, Hand M, VanBracle L, McVay F. Validation of a predictive model to identify patients at high risk for hospital admission. J Healthc Qual 2016;38(1):34-31.

25. Wang H, Robinson RD, Johnson C, Zenarosa NR, Jayswal RD, Keithley J, Delaney KA. Using the LACE index to predict hospital readmissions in congestive heart failure patients. BMC Cardiovasc Disord 2014;14:97.

26. Cotter P, Bhalla VK, Wallis SJ, Biram RWS. Predicting readmissions: poor performance of the LACE index in an older UK population. Age Ageing 2012;41(6):784-9.

27. Kreilkamp, R. Application of the LACE risk assessment tool at Chinese hospital. The Advisory Board Group. http://www.avoidreadmissions.com/wwwroot/userfiles/documents/55/lace-risk-assessmenttool.pdf. Published 2011. Accessed 1st March 2014.

28. Tong L, Erdmann C, Daldalian M, Li J, Esposito T. Comparison of predictive modelling approaches for 30-day all-cause non-elective readmission risk. BMC Med Res Methodol 2016;16:26.

29. Yazaan-Ashoori P, Lee SK, Ibrahim Q, Van Spall HG. Utility of the LACE index at the bedside in predicting 30 day readmission or death in patients hospitalized with heart failure. Am Heart J 2016;179:51-8.

30. Bashir B, Schneider D, Naglak MC, Churilla TM, Adelsherger M. Evaluation of prediction strategy and care coordination for COPD readmissions. Hosp Pract 2016;44(3):123-8.

31. Mixon AS, Goggins K, Bell SP, Vasilevskis EE, Nwosu S, Schildcrout JS, Kripalani S. Preparedness for hospital discharge and prediction of readmission. J Hosp Med 2016;11(9):603-9.

32. Clarke A. Are readmissions avoidable? BMJ 1990;301:1136-8.

For peer review only - http://bmjopen.bmj.com/site/about/guidelines.xhtml

For peer review only

33. Drozda JP. Readmission rates. BMJ 2013;347.

34. Blunt I, Bardsley M, Dixon J. Trends in emergency admissions in England 2004-2009. Nuffield Trust, London: 2010.

35. Blunt I, Bardsley M, Grove A, Clarke A. Classifying emergency 30 day readmissions in England using routine hospital data 2004-2010: what is the scope for reduction? Emerg Med J 2014.

36. Billings J, Blunt I, Steventon A, Gheorghiou T, Lewis G, Bardsley M. Development of a predictive model to identify inpatients at risk of re-admission within 30 days of discharge (PARR-30). BMJ Open 2012; 00:e001667.

37. Van Walraven C, Wong J, Forster AJ. LACE+ index: extension of a validated index to predict early death or urgent readmission after hospital discharge using administrative data. Open Medicine 2012; 6(3):e90.

38. Donze JD, Williams MV, Robinson EJ, Zimlichman E, Avjesky D, Vasilevskis EE, Kripalani S, Metlay JP, Wallington T, Fletcher GS, Averbach AD, Schnipper JL. International validity of the HOSPITAL score to predict 30-day potentially avoidable readmission. JAMA Intern Med 2016;176(4):496-502.

39. Low LL, Liu N, Wang S, Thumboo J, Ong ME, Lee KH. Predicting 30 day readmission in an Asian population: building a predictive model by incorporating markers of hospitalization severity. PLoS One 2016;11(2):e0167413.

40. Garrison GM, Robelia PM, Pecina JL, Dawson NL. Comparing performance of 30-day readmission risk classifiers among hospitalized primary care patients. J Eval Clin Pract 2016;Epub ahead of print.

41. Szekendi MK, Williams MV, Carrier D, Hensley L, Thomas S, Cerese J. The characteristics of patients frequently admitted to academic medicine centers in the United States. J Hosp Med 2015;10(9):563-8.

For peer review only

For peer review only - http://bmjopen.bmj.com/site/about/guidelines.xhtml

STROBE 2007 (v4) Statement—Checklist of items that should be included in reports of *cohort studies*

Section/Topic	Item #	Recommendation	Reported on page #
**Title and abstract**	1	(*a*) Indicate the study's design with a commonly used term in the title or the abstract	1
		(*b*) Provide in the abstract an informative and balanced summary of what was done and what was found	2
**Introduction**			
Background/rationale	2	Explain the scientific background and rationale for the investigation being reported	4
Objectives	3	State specific objectives, including any prespecified hypotheses	4
**Methods**			
Study design	4	Present key elements of study design early in the paper	4-5
Setting	5	Describe the setting, locations, and relevant dates, including periods of recruitment, exposure, follow-up, and data collection	4-5
Participants	6	(*a*) Give the eligibility criteria, and the sources and methods of selection of participants. Describe methods of follow-up	4-5
		(*b*) For matched studies, give matching criteria and number of exposed and unexposed	N/A
Variables	7	Clearly define all outcomes, exposures, predictors, potential confounders, and effect modifiers. Give diagnostic criteria, if applicable	5-6
Data sources/ measurement	8*	For each variable of interest, give sources of data and details of methods of assessment (measurement). Describe comparability of assessment methods if there is more than one group	4-5
Bias	9	Describe any efforts to address potential sources of bias	N/A
Study size	10	Explain how the study size was arrived at	N/A: all patients in a two-year period were included
Quantitative variables	11	Explain how quantitative variables were handled in the analyses. If applicable, describe which groupings were chosen and why	6
Statistical methods	12	(*a*) Describe all statistical methods, including those used to control for confounding	6
		(*b*) Describe any methods used to examine subgroups and interactions	6
		(*c*) Explain how missing data were addressed	6
		(*d*) If applicable, explain how loss to follow-up was addressed	N/A

For peer review only

		(*e*) Describe any sensitivity analyses	N/A
**Results**			
Participants	13*	(a) Report numbers of individuals at each stage of study—eg numbers potentially eligible, examined for eligibility, confirmed eligible, included in the study, completing follow-up, and analysed	N/A: all patients in a two-year period were included
		(b) Give reasons for non-participation at each stage	N/A
		(c) Consider use of a flow diagram	N/A
Descriptive data	14*	(a) Give characteristics of study participants (eg demographic, clinical, social) and information on exposures and potential confounders	6
		(b) Indicate number of participants with missing data for each variable of interest	N/A: patient episodes with missing data were removed from the dataset
		(c) Summarise follow-up time (eg, average and total amount)	N/A
Outcome data	15*	Report numbers of outcome events or summary measures over time	6-7
Main results	16	(*a*) Give unadjusted estimates and, if applicable, confounder-adjusted estimates and their precision (eg, 95% confidence interval). Make clear which confounders were adjusted for and why they were included	6-9
		(*b*) Report category boundaries when continuous variables were categorized	6-9
		(*c*) If relevant, consider translating estimates of relative risk into absolute risk for a meaningful time period	N/A
Other analyses	17	Report other analyses done—eg analyses of subgroups and interactions, and sensitivity analyses	6-9
**Discussion**			
Key results	18	Summarise key results with reference to study objectives	9
**Limitations**			
Interpretation	20	Give a cautious overall interpretation of results considering objectives, limitations, multiplicity of analyses, results from similar studies, and other relevant evidence	10
Generalisability	21	Discuss the generalisability (external validity) of the study results	10-11
**Other information**			
Funding	22	Give the source of funding and the role of the funders for the present study and, if applicable, for the original study on which the present article is based	12

For peer review only

*Give information separately for cases and controls in case-control studies and, if applicable, for exposed and unexposed groups in cohort and cross-sectional studies.

Note: An Explanation and Elaboration article discusses each checklist item and gives methodological background and published examples of transparent reporting. The STROBE checklist is best used in conjunction with this article (freely available on the Web sites of PLoS Medicine at http://www.plosmedicine.org/, Annals of Internal Medicine at http://www.annals.org/, and Epidemiology at http://www.epidem.com/). Information on the STROBE Initiative is available at www.strobe-statement.org.

For peer review only

Evaluating the predictive strength of the LACE index in identifying patients at high risk of hospital readmission following an inpatient episode: retrospective cohort study

Journal:	*BMJ Open*
Manuscript ID	bmjopen-2017-016921.R1
Article Type:	Research
Date Submitted by the Author:	04-May-2017
Complete List of Authors:	Damery, Sarah; University of Birmingham, Primary Care Clinical Sciences<br>Combes, Gill; University of Birmingham, Institute of Applied Health Research
**Primary Subject Heading**:	Health policy
Secondary Subject Heading:	Evidence based practice
Keywords:	LACE, readmissions, case finding, risk stratification, hospital

SCHOLARONE™ Manuscripts

For peer review only

Evaluating the predictive strength of the LACE index in identifying patients at high risk of hospital readmission following an inpatient episode: retrospective cohort study

Dr Sarah Damery, Research Fellow, Institute of Applied Health Research, College of Medical and Dental Sciences, University of Birmingham, Edgbaston, West Midlands, UK, B15 2TT

Dr Gill Combes, CLAHRC West Midlands Research Lead for Chronic Diseases Theme, Institute of Applied Health Research, College of Medical and Dental Sciences, University of Birmingham, Edgbaston, West Midlands, UK, B15 2TT

Corresponding author:
Dr Sarah Damery
Research Fellow
Institute of Applied Health Research
College of Medical and Dental Sciences
University of Birmingham
Edgbaston
West Midlands
UK
B15 2TT

Email: s.l.damery@bham.ac.uk

For peer review only

Abstract

Objective:

To assess how well the LACE index and its constituent elements predicts 30-day hospital readmission and to determine whether other combinations of clinical or sociodemographic variables may enhance prognostic capability.

Design:

Retrospective cohort study with split sample design for model validation.

Setting:

One large hospital Trust in the West Midlands.

Participants:

All alive-discharge adult inpatient episodes between 1st January 2013 and 31st December 2014.

Data sources:

Anonymised data for each inpatient episode were obtained from the hospital information system. These included age at index admission, gender, ethnicity, admission/discharge date, length of stay, treatment specialty, admission type and source, discharge destination, comorbidities, number of accident and emergency (A&E) visits in the six months before the index admission, and whether a patient was readmitted within 30 days of index discharge.

Outcome measures:

Clinical and patient characteristics of readmission vs. non-readmission episodes, proportion of readmission episodes at each LACE score, regression modelling of variables associated with readmission to assess the effectiveness of LACE and other variable combinations to predict 30-day readmission.

Results:

The training cohort included data on 91,922 patient episodes. Increasing LACE score and each of its individual components were independent predictors of readmission (AUC 0.773; 95% CI: 0.768 to 0.779 for LACE; AUC 0.806; 95% CI: 0.801 to 0.812 for the four LACE components). A LACE score of 11 was most effective at distinguishing between higher and lower risk patients. However, only 25% of readmission episodes occurred in the higher scoring group. A model combining A&E visits and hospital episodes per patient in the previous year was more effective at predicting readmission (AUC 0.815; 95% CI: 0.810 to 0.819).

Conclusions:

Although LACE shows good discriminatory power in statistical terms, it may have little added value over and above clinical judgement in predicting a patient's risk of hospital readmission.

Word count:

3164

Keywords: LACE, readmissions, case finding, risk stratification, hospital

For peer review only

STRENGTHS AND LIMITATIONS OF THIS STUDY

- This study assessed the characteristics associated with 30-day hospital readmission in a large hospital Trust in the West Midlands
- A split sample design allowed mode development and statistical testing to be undertaken in one half of the dataset and the results validated in a representative sample of inpatient episodes from a directly comparable population
- In focusing on a general medical population, the study evaluated the LACE index in a context similar to that in which it was originally developed
- Readmission rates may have been underestimated as we were unable to identify cases where a patient may have been readmitted to another hospital

For peer review only

For peer review only - http://bmjopen.bmj.com/site/about/guidelines.xhtml

INTRODUCTION

In recent years, developing effective ways to reduce rates of patient readmission following an episode of acute care has become a key health policy focus in many developed economies.[1] In 2011/12, the average 30-day readmission rate in England was 5.6%, with variation across acute Trusts of between 3 and 10%.[2] It is estimated that 30-day readmissions incur annual costs in excess of £2.5 billion for the National Health Service (NHS),[3] and in 2011, the Department of Health introduced a policy of non-payment to hospitals in England for emergency readmissions within 30 days of discharge following an elective admission. In 2012, this was extended to encompass both elective and emergency admissions. The policy applies to all clinical areas except those where it is considered inappropriate to withhold payment (e.g. readmission in cancer or dialysis patients), and operates by establishing local readmission thresholds following clinical review of readmissions at a given Trust and determining the proportion that could be considered avoidable.[4] In a financially straitened NHS, the prospect of incurring financial penalties for 30-day readmissions has created a strong incentive for hospital Trusts to reduce readmission rates.

The most common clinical reasons for readmission are infections, and complications related to medical care or long term conditions,[5] and it is thought that readmission rates can be reduced substantially if at-risk patients can be identified before discharge and offered supportive interventions as inpatients or after discharge. However, interventions are most likely to be effective if they are targeted towards patients at highest risk of future hospital use,[6] as being able to distinguish between patients who will not require readmission and those who are likely to be readmitted has implications for the cost-effectiveness of readmission avoidance interventions.[7-9] Identifying at-risk patients effectively relies on accurate case finding,[10] and a large number of predictive models have been used both within the NHS,[3,11-13] and internationally,[14-16] to varying degrees of success.[17-19] Predictive models differ in the type and scope of data items they include and the time period over which they seek to predict readmission risk.[20] When choosing an appropriate model, a trade-off often needs to be made between complexity - the number of data items required - and practicality of application in clinical practice.[21]

One widely used predictive tool is the LACE index,[22] which was originally developed in Canada and uses routinely collected clinical and administrative data to generate a risk score of between 0 and 19 for individual patients, where higher scores indicate an increased risk of readmission. Scores are based on four features of an inpatient hospital episode: length of stay, admission type, comorbidities and the number of Accident and Emergency (A&E) visits made by a patient in the six months prior to their initial admission. Scores over a specific threshold can be used to 'flag' at-risk patients for whom interventions may be appropriate. Although widely used – largely due to its simplicity and the ease of LACE score calculation using data routinely collected by all hospital Trusts, the evidence base for LACE is uncertain. Some studies have found it to be an effective predictor of readmission,[23,24] whereas others have demonstrated poor prognostic ability, particularly when applied to specific patient sub-groups.[25,26] The literature on case finding tools emphasises the importance of local validation before implementation, since each hospital has a patient case mix that reflects their surrounding population and may require a locally-calibrated score threshold.[13] A modified version of the LACE index has been developed,[27] that gives greater weight to patient comorbidities, which are considered a key driver of readmissions.[21] This study analysed data from a large hospital Trust in the West Midlands to assess how well the (modified) LACE index and each of its constituent elements predicts 30-day readmission and to determine whether a model based on other combinations of clinical or patient variables may enhance prognostic capability.

For peer review only - http://bmjopen.bmj.com/site/about/guidelines.xhtml

METHODS

Sampling

The study used a retrospective cohort design with a split sample to allow the findings to be externally validated. All alive-discharge adult inpatient episodes at a large hospital Trust in the West Midlands over a two year period (1st January 2013 to 31st December 2014) were included in the analysis. Data were obtained following a search of the Trust information system performed by the Trust IT manager. Anonymised sociodemographic and clinical data were obtained for each inpatient episode (termed the 'index admission') . Sociodemographic data included patient age at index admission, gender and ethnic group. Clinical data relating to the index admission included: date of admission and discharge, length of stay (LoS), ICD10 code, primary diagnosis, treatment specialty, Health Research Group (HRG) code, admission type (emergency, elective, day case), admission source and discharge destination (e.g. usual place of residence, other NHS institution). Data were also obtained on patient comorbidities, number of A&E visits in the six months before the index admission, whether the index episode was followed by readmission within 30 days of the index discharge date, and if so, the date of readmission and treatment specialty.

Data analysis

A LACE score was calculated based on LoS (0 to 6 points), admission type (0 to 3 points), comorbidity (0 to 6 points) and previous A&E attendance (0 to 4 points), giving a total score between 0 and 19 for each inpatient episode (Table 1).

Table 1: Components of the modified LACE index and values assigned for each

Attribute	Value	Points
Length of stay	Less than 1 day	0
	1 day	1
	2 days	2
	3 days	3
	4 to 6 days	4
	7 to 13 days	5
	14 or more days	6
Acute admission	Inpatient	3
	Observation	0
Comorbidity (scores cumulative to a maximum of 6)	No prior history	0
	Diabetes without complications, cerebrovascular disease, history of myocardial infarction (MI), peripheral vascular disease (PVD), peptic ulcer disease (PUD)	1
	Mild liver disease, diabetes with end organ damage, congestive heart failure (CHF), chronic obstructive pulmonary disease (COPD), cancer, leukaemia, lymphoma, any tumour, cancer, moderate to severe renal disease	2
	Dementia or connective tissue disease	3
	Moderate/severe liver disease or human immunodeficiency virus (HIV) infection	4
	Metastatic cancer	6
A&E visits during previous 6 months	0 visits	0
	1 visits	1
	2 visits	2
	3 visits	3
	4 or more visits	4

For peer review only - http://bmjopen.bmj.com/site/about/guidelines.xhtml

These scores differ from the original LACE index[22] in two ways. First, the original LACE index assigns up to 7 points for a length of stay lasting 14 or more days, whereas the modified LACE index gives up to 6 points for this parameter. Second, the comorbidity element of the original LACE index is scored up to a maximum of 5, whereas the modified LACE index allows comorbidity scores up to 6 points.

Any patient episodes with missing data were removed from the dataset (n=4,503 episodes; 2.4% of the total). Missing data items fell into three groups: i) patient was discharged from their index hospital episode after 31st December 2014 (n=727), ii) no date of discharge from index hospital episode was available (n=661), and iii) patient died during their index admission (n=3,115). After removal of records with missing data, the dataset was split in half at random to create a cohort for model building (the ‘training cohort’) and a separate cohort for model validation (the ‘test cohort’). As a split sample design was used to derive the two cohorts from the same original dataset (ensuring patient and clinical profiles were directly comparable across the cohorts and minimising the likelihood of model over fitting), internal cross-validation within the training cohort was not performed during model development.

Normality testing indicated that the continuous data were not normally distributed. As a result, all continuous variables were summarised using medians and interquartile ranges (IQR) and univariate comparisons of these variables across the readmitted/non-readmitted groups used the non-parametric Mann-Whitney *U*-test. Chi-squared tests were used to compare the characteristics of readmitted vs. non-readmitted patients for variables with categorical data. Univariate odds ratios (OR) and their 95% confidence intervals (CI) were calculated for sociodemographic and clinical variables and for each component of the LACE index to test the association between variable subgroups and readmission. Finally, binary logistic regression modelling using the enter method was used to test the strength of different combinations of variables in predicting the likelihood of 30 day readmission. Model strength was described using OR, and the area under the receiver operating characteristic (ROC) curve described using the *c*-statistic. The findings for each model were then validated using the patient episodes in the test cohort. All statistical analyses were undertaken using SPSS (version 21, SPSS Inc., Chicago IL).

RESULTS

Sample characteristics

The full dataset included 183843 patient episodes (103493 individual patients). After splitting the dataset to create the training and test cohorts, subsequent analyses were performed on the training cohort prior to validation, which contained data on 91,922 separate admission episodes (representing 51747 individual patients) (Table 2).

For peer review only - http://bmjopen.bmj.com/site/about/guidelines.xhtml

Table 2: Characteristics of readmission vs. non-readmission episodes

Variable	Grouping	Total episodes (%)	Readmitted (%)	Not readmitted (%)	Comparison*
Patient age	Median, range (IQR)	55.0, 18 to 106 (37 to 72)	64.0, 18 to 105 (44 to 78)	55.0, 18 to 106 (37 to 71)	p<0.0001
Gender	Male	39001 (42.4)	3545 (9.1)	35456 (90.9)	X^2=175.1; p<0.0001
	Female	52921 (57.6)	3562 (6.7)	49359 (93.3)	
Index LoS (days)	Median, range (IQR)	0.0, 0 to 301 (0.0 to 2.0)	1.0, 0 to 223 (0.0 to 5.0)	0.0, 0 to 301 (0.0 to 1.0)	p<0.0001
Admission type	Emergency	46922 (51.0)	6005 (12.8)	40917 (87.2)	X^2=3573.4; p<0.0001
	Elective	7243 (7.9)	410 (5.7)	6833 (94.3)	
	Day case	37757 (41.1)	692 (1.8)	37065 (98.2)	
Comorbidity score**	0	74274 (80.8)	5083 (6.8)	69191 (93.2)	X^2=1126.9; p<0.0001
	1	14984 (16.3)	1820 (12.1)	13164 (87.9)	
	2	2147 (2.3)	29 (1.4)	2118 (98.6)	
	3	514 (0.6)	172 (33.5)	342 (66.5)	
	4	3 (0.0)	3 (100.0)	0 (0.0)	
	6	0 (0.0	0 (0.0)	0 (0.0)	
A&E visits in previous 6 months	Median, range (IQR)	1.0, 1 to 121 (1.0 to 2.0)	2.0, 1 to 107 (1.0 to 4.0)	1.0, 1 to 121 (1.0 to 2.0)	p<0.0001

* Continuous variables were compared using the Mann-Whitney *U*-test and categorical variables were compared using the X^2 test. ** Comorbidity score does not relate to number of comorbidities; scores are assigned based on severity of comorbidities

Median patient age in the training cohort was 55 (IQR: 37 to 72), and male patients accounted for 42.4% of hospital episodes (n=39001). The median LoS of the index admission was 0 days (IQR: 0 to 2). 51.0% of episodes followed emergency admission (n=46922). The majority of patients had a comorbidity score of 0 (80.8%) and the median number of A&E visits in the 6 months prior to the index episode was 1 (IQR: 0 to 1).

Characteristics of readmitted vs. non-readmitted patients

A total of 7107 inpatient episodes were followed by a readmission within 30 days (7.7% readmission rate, 4541 individual patients). 1218 patients (2.4%) accounted for 53.1% of all readmission episodes. A comparison of the characteristics of episodes that resulted in readmission vs. those that did not showed statistically significant differences for all variables. Readmitted patients were significantly more likely to be older than those who were not readmitted (median age 64 vs. 55), men had significantly higher readmission rates than women (9.1% vs. 6.7%), and emergency admissions were significantly more likely to result in readmission than elective or day case admissions (12.8% vs. 5.7% and 1.8% respectively). Median LoS in readmitted patients was 1 day (IQR: 0 to 5); median A&E visits in the previous 6 months was 2 (IQR: 1 to 4), and higher comorbidity scores were significantly associated with readmission, with 33.5% of patients with a comorbidity score of 3 being readmitted within 30 days.

Readmission episodes and LACE score

The median LACE score in the training cohort was 5 (range 3 to 17, IQR: 3 to 7) (Table 3). The Mann-Whitney U-test showed that the median score for readmission episodes was significantly higher than the median score for episodes that did not result in readmission (8.0 with IQR 6 to 11 vs. 5.0 with IQR 3 to 7; p<0.0001). Readmission rates more than doubled between a LACE score of 10 and 11, suggesting that 11 may be the optimum threshold for distinguishing between patients at a lower or higher risk of readmission. However, the proportion of total readmissions represented by LACE scores 11 and above was only 25.3% of

For peer review only

For peer review only - http://bmjopen.bmj.com/site/about/guidelines.xhtml

the total (1795/7107), thus nearly three quarters of readmissions occurred in patients scoring lower than the cut-off point.

Table 3: Proportion of readmission episodes at each LACE score

LACE score*	Total episodes (%)	Readmitted (%)	Not readmitted (%)
3	26478 (28.8)	302 (1.1)	26176 (98.9)
4	13798 (15.0)	561 (4.1)	13237 (95.9)
5	14152 (15.4)	676 (4.8)	13476 (95.2)
6	10656 (11.6)	753 (7.1)	9903 (92.9)
7	8637 (9.4)	1045 (12.1)	7592 (87.9)
8	5800 (6.3)	913 (15.7)	4887 (84.3)
9	4136 (4.5)	551 (13.3)	3585 (86.7)
10	3242 (3.5)	511 (15.8)	2731 (84.2)
11	2379 (2.6)	815 (34.3)	1564 (65.7)
12	1570 (1.7)	633 (40.3)	937 (59.7)
13	751 (0.8)	231 (30.8)	520 (69.2)
14	217 (0.2)	81 (37.3)	136 (62.7)
15	104 (0.1)	35 (33.7)	69 (66.3)
16	1 (0.0)	0 (0.0)	1 (100.0)
17	1 (0.0)	0 (0.0)	1 (100.0)

* Percentages for readmitted and not readmitted are calculated according to variable grouping e.g. % of episodes scoring 3 on the LACE index which resulted in readmission vs. those that did not

Univariate logistic regression

Univariate binary logistic regression assessed the association between individual variables and the likelihood of readmission (Table 4). All variables were statistically significant to the $p<0.0001$ level. For each unit increase in patient age, the likelihood of readmission rose by 1.7%. Females were significantly less likely to be readmitted than males, despite constituting a larger proportion of index admissions (OR 0.72; 95% CI: 0.69 to 0.76). Increasing LACE score was significantly associated with 30 day readmission, with each point increase in score associated with a 42% increase in the likelihood of readmission. The variable with the strongest association with readmission was emergency admission – index episodes which were a result of emergency admission were nearly 8 times more likely to be followed by readmission than those in the reference group of day case surgery (OR 7.87; 95% CI: 7.26 to 8.52).

For peer review only - http://bmjopen.bmj.com/site/about/guidelines.xhtml

Table 4: Univariate logistic regression of variables potentially associated with readmission

Variable	Grouping	P value	Odds ratio (95% CI)
Patient age	Continuous	<0.0001	1.03 (1.02 to 1.04)
Patient gender	Male	Reference	Reference
	Female	<0.0001	0.72 (0.69 to 0.76)
Length of stay	Continuous	<0.0001	1.04 (1.03 to 1.04)
Admission type	Day case	Reference	Reference
	Elective	<0.0001	3.21 (2.84 to 3.64)
	Emergency	<0.0001	7.87 (7.26 to 8.52)
Comorbidity score	Continuous	<0.0001	1.28 (1.25 to 1.31)
A&E visits in previous 6 months	Continuous	<0.0001	1.39 (1.38 to 1.41)
LACE score	Continuous	<0.0001	1.42 (1.41 to 1.43)
Episodes per patient in previous year	Continuous	<0.0001	1.06 (1.05 to 1.06)

Multivariate logistic regression

Four multivariate logistic regression models were constructed to assess different potential predictors of 30 day readmission (Table 5). The first included LACE index score only. LACE score was highly significant as a predictor of readmission (p<0.0001), with an AUC of 0.773 (95% CI: 0.768 to 0.779) and R^2 of 0.180. This is higher than the *c*-statistic for LACE score as a predictor of readmission found by van Walraven et al who developed the LACE index. However, the other three models all had a higher *c*-statistic: model 2, which included all eight variables from the univariate analysis had a *c*-statistic of 0.820 (95% CI: 0.815 to 0.825) and R^2 of 0.240. In this model, whilst all included variables were significant at the p<0.0001 level, the direction of effect for some variables differed from the univariate testing. Both increasing length of stay and higher comorbidity score were associated with a *lower* likelihood of readmission in this model when the effects of other variables was controlled for. This is likely to be due to an association between patient age and/or gender with comorbidities and length of stay. Model 3 included the four components of the LACE index, and was a better predictor of readmission than LACE score alone, with a *c*-statistic of 0.806 (0.801 to 0.812). In the fourth model, which included only A&E visits and number of episodes per patient as variables, was a better model for predicting readmission than models 1 and 3 and had only a marginally lower *c*-statistic than model 2 which was the most complex in terms of number of variables included (AUC=0.815; 95% CI: 0.810 to 0.819).

Model validation

The models developed using the training cohort were tested for validity in the test cohort. The test cohort did not differ from the training cohort in any of the sociodemographic of clinical variables assessed. It included 91921 episodes of care, of which 7008 (7.6%) were followed by a readmission within 30 days. The *c*-statistic of the logistic regression models developed from the training cohort were 0.767 (0.761 to 0.772) for model 1, 0.814 (0.809 to 0.189) for model 2, 0.800 (0.794 to 0.805) for model 3 and 0.812 (0.807 to 0.817) for model 4.

For peer review only - http://bmjopen.bmj.com/site/about/guidelines.xhtml

Table 5: Binary logistic regression models assessing the probability of readmission

Variable	P value	Odds ratio (95% CI)	AUC (95% CI); R^2
Model 1: LACE score only			
LACE score	<0.0001	1.42 (1.41 to 1.43)	AUC=0.773 (0.768 to 0.779); R^2=0.180
Model 2: All variables from univariate analysis			
Age	<0.0001	1.01 (1.01 to 1.02)	AUC=0.820 (0.815 to 0.825); R^2=0.240
Gender (female)	<0.0001	0.90 (0.85 to 0.95)	
Length of stay	<0.0001	0.98 (0.98 to 0.99)	
Admission type (emergency)	<0.0001	4.18 (3.77 to 4.64)	
Comorbidity score	<0.0001	0.96 (0.94 to 0.98)	
A&E visits in previous 6 months	<0.0001	1.12 (1.11 to 1.13)	
LACE score	<0.0001	1.23 (1.21 to 1.25)	
Episodes per patient	<0.0001	1.07 (1.06 to 1.07)	
Model 3: Four components of the LACE index			
Length of stay	<0.0001	1.02 (1.01 to 1.03)	AUC=0.806 (0.801 to 0.812); R^2=0.193
Admission type (emergency)	<0.0001	4.79 (4.40 to 5.22)	
Comorbidity score	<0.0001	1.30 (1.26 to 1.33)	
A&E visits in previous 6 months	<0.0001	1.28 (1.27 to 1.29)	
Model 4: Reduced complexity model			
A&E visits in previous 6 months	<0.0001	1.36 (1.35 to 1.38)	AUC=0.815 (0.810 to 0.819); R^2=0.130
Episodes per patient in previous year	<0.0001	1.03 (1.02 to 1.04)	

DISCUSSION

The primary aim of this study was to assess how well the modified LACE index was able to predict 30-day readmission in a cohort of patients admitted to a large secondary care Trust over a two year period. Increasing LACE score and the four individual components comprising the LACE index were all independent predictors of readmission. The proportion of admissions episodes resulting in readmission increased substantially at a LACE score cut-off of 11, which would suggest that this is an appropriate threshold to use when deciding whether to provide enhanced inpatient and/or post-discharge care to prevent unplanned readmission. However, although a large proportion of admissions episodes that scored 11+ on the LACE index were followed by a readmission within 30 days, this corresponded to comparatively few absolute numbers of patients. Only 25% of all readmissions occurred in the higher scoring group, whilst the remaining 75% occurred following episodes of care that scored <11 on the index. This differs from other studies that have assessed the effectiveness of different risk thresholds for LACE, which typically saw a higher proportion of all readmissions occurring in the patient group that scored above the chosen threshold.[23,28] Whilst implementing a lower LACE score threshold would improve the likelihood of identifying at-risk patients, a large number of these patients would not go on to be readmitted. In a health service facing substantial resource constraints, the LACE tool is unlikely to have the sensitivity and specificity that would make it a useful addition to clinical practice.

A number of studies have assessed the performance on the LACE index in predicting unplanned readmissions but these have typically been conducted in small patient populations,[29,30] or in specific patient groups such as cardiovascular disease,[25,29,31] COPD,[30] or older people.[26] The patient cohort included in this

For peer review only

study was large, and the analysis had good statistical power to detect differences between groups. In focusing on a general medical population, our study evaluated the LACE index in a context similar to that in which it was originally developed in terms of patient characteristics and incidence of comorbidity.[22,27] The split sample design allowed model development and statistical testing to be carried out in one half of the dataset, and the results validated in a representative sample of inpatient episodes from a directly comparable population. Readmission rates may have been underestimated in the hospital data used for this study, as we were unable to identify instances where a discharged patient may have subsequently been readmitted to another hospital. Patient deaths were not recorded in the data (unless a patient died during their index admission), so we were unable to consider the impact of patient mortality on our findings.

Multiple factors typically contribute to readmission rates, and there are limits on the extent to which unplanned readmissions can be avoided.[32,33] High readmission rates are often thought to indicate sub-optimal patient management, but they are most likely to be driven by difficulties in managing patient transitions to other health and social care settings, a lack of community resources for patient follow-up, or influenced by the patient's home environment.[34] A retrospective analysis of 82 million routinely collected hospital records in England between 2004-2010 found that only 30% of unplanned readmissions were deemed avoidable.[35] Therefore, lowering readmission rates for patients with chronic or relapsing conditions, or patients readmitted with a different diagnosis from their index admission, poses a significant challenge. Conversely, avoiding readmissions in patients presenting with a recurrence or continuation of the issue that led to their initial hospitalisation, or for those who are readmitted with an avoidable complication related to their index admission should be a priority for hospital Trusts, which is a key reason that case finding tools are increasingly being tested in the hospital setting. Although a number of increasingly complex tools have been developed in recent years, such as PARR-30,[36] LACE+,[37] and HOSPITAL,[38] the intuitive appeal of LACE lies in its simplicity and use of routinely collected hospital data.

This study suggests that despite a number of sociodemographic and clinical variables being strongly associated with hospital readmission in statistical terms, the added value of the LACE tool over and above clinical judgement remains equivocal. However, the fact that small gains in model accuracy and discriminatory power can be made by testing different combinations of potential predictor variables derived from routinely collected hospital administrative data may indicate that the accuracy of case finding could be improved through the addition of locally-relevant clinical or sociodemographic factors.[39,40] In this study, the predictive model with the least discriminatory power was based on LACE score alone. Model 2, which included eight predictor variables, was only marginally better at predicting readmission than model 4 which included only two variables: A&E visits and the number of admissions per patient in the previous 12 months. This would suggest – in a cohort of general medical admissions - that a simpler model could outperform the more complex LACE tool in accurately identifying patients at risk of readmission. Our analysis showed that 2.4% of patients in the cohort accounted for 53.1% of all readmission episodes. Being able to identify the small group of patients who use a disproportionate amount of healthcare resources is the first step towards developing solutions to prevent repeat hospitalisations in this population.[41] Future research should focus on the development of locally-tailored screening tools to identify these patients.

CONCLUSION

Although LACE shows good discriminatory power in statistical terms, it may have little added value over and above clinical judgement in predicting a patient's risk of hospital readmission. Nevertheless, if used as a screening tool alongside clinical judgement, a locally-tailored risk score based on specific clinical or sociodemographic variables relevant to the inpatient population admitted to a particular hospital Trust may increase case finding accuracy. This could allow clinicians to effectively discriminate between patients who are likely to have an unplanned admission within 30 days of discharge and those that will not.

For peer review only - http://bmjopen.bmj.com/site/about/guidelines.xhtml

DECLARATIONS

Declaration of competing interests: All authors have completed the Unified Competing interest form at www.icmje.org/coi_disclosure.pdf (available on request from the corresponding author) and declare no support from any organisations for the submitted work; no financial relationships with any organisations that might have an interest in the submitted work in the previous three years; no other relationships or activities that could appear to have influenced the submitted work.

Acknowledgements: We would like to thank Martin Chadderton for providing the data from the hospital information system, and Roger Steadman for clinical guidance on the study.

Details of contributors: SD and GC designed the study. SD wrote the study protocol. SD undertook data analysis, with input from GC as needed. SD drafted and revised the paper and is guarantor for the work. GC critically revised the paper for intellectual content. All authors gave final approval of the manuscript and are accountable for all aspects of the accuracy and integrity of the work.

Ethics approval: Ethical approval was obtained from the University of Birmingham Research Ethics Committee (Ref: ERN_14-0914). Research governance approval was obtained from the R&D office of Sandwell and West Birmingham Hospitals NHS Trust (Ref: 14MISC40).

Funding: This research was funded by the National Institute of Health Research (NIHR) Collaboration for Leadership in Applied Health Research and Care West Midlands (CLAHRCWM).

Role of study sponsor and funder: The study sponsor and funder had no role in the study design, in the collection, analysis and interpretation of data, in the writing of the report, and in the decision to submit the article for publication.

Independence from funders: This paper presents independent research funded by the National Institute of Health Research (NIHR) Collaboration for Leadership in Applied Health Research and Care West Midlands (CLAHRCWM). The views expressed are those of the authors and not necessarily those of the NHS, the NIHR or the Department of Health.

Data access: All authors had full access to all of the data (including statistical reports and tables) in the study and can take responsibility for the integrity of the data and the accuracy of the data analysis.

Transparency: The guarantor (SD) affirms that the manuscript is an honest, accurate and transparent account of the study being reported; no important aspects of the study have been omitted, and any discrepancies from the study as planned and registered have been explained.

Data sharing: No additional data available.

For peer review only

REFERENCES

1. Conroy S, Dowsing T. What should we do about hospital readmissions? Age Ageing 2012;41(6):702-4.

2. SG2. Reducing 30-day readmissions. SG2 Service kit; June 2011. https://www.hsj.co.uk/Journals/2/Files/2011/6/15/Sg2_Service%20Kit_Reducing%2030-Day%20Readmissions.pdf. Accessed 15th March 2014.

3. Billings J, Blunt I, Steventon A, Gheorghiou T, Lewis G, Bardsley M. Development of a predictive model to identify inpatients at risk of re-admission within 30 days of discharge (PARR-30). BMJ Open 2012;00:e001667.

4. Monitor and NHS England. 2016/17 National Tariff Payment System. Monitor, 2016; London.

5. Robinson P. Hospital readmissions and the 30 day threshold. CHKS Marketing Intelligence Report, 2016; London.

6. Ross S, Curry N, Goodwin N. Case management: what it is and how it can best be implemented. The Kings Fund, London: 2011.

7. Purdy S. Avoiding hospital admissions. What does the research evidence say? The King's Fund, London; 2010.

8. Dixon J, Bardsley M. Predictive risk modelling using routine data: underexploited potential to benefit patients. Journal of Health Services Research Policy 2012;17(3):131-2.

9. Billings J, Gheorghiou T, Blunt I, Bardsley M. Choosing a model to predict hospital admission: an observational study of new variants of predictive models for case finding. BMJ Open 2013;3:e003352.

10. Lewis G, Curry N, Bardsley M. How predictive modelling can help reduce risk and hospital admissions. Nuffield Trust, London: 2011.

11. Donnan PT, Dorward DWT, Mutch B, Morris AD. Development and validation of a model for predicting emergency admissions over the next year (PEONY). Arch Intern Med 2008;168(13):1416-22.

12. Chenore T, Pereira Gray DJ, Forrer J, Wright C, Evans PH. Emergency hospital admissions for the elderly: insights from the Devon Predictive Model. Journal of Public Health 2013;35(4):616-23.

13. Gheorghiou T, Billings J, Bardsley M. New predictive case finding models for the NHS. Nuffield Trust, London: 2013.

14. Halfon P, Eggli Y, Pretre-Rohrbach I, Meylan D, Marazzi A, Burnand B. Validation of the potentially avoidable hospital readmission rate as a routine indicator of the quality of hospital care. Med Care 2006;44(11):972-81.

15. Silverstein MD, Qin H, Mercer SQ, Fong J, Haydar Z. Risk factors for 30-day hospital readmission in patients >65 years of age. Proc (Bayl Univ Med Cent) 2008;21(4):363-72.

16. Howell S, Coory M, Martin J, Duckett S. Using routine inpatient data to identify patients at risk of hospital admission. BMC Health Serv Res 2009;9:96.

For peer review only - http://bmjopen.bmj.com/site/about/guidelines.xhtml

17. Hansen LO, Young RS, Hinami K, Leung A, Williams MV. Interventions to reduce 30-day hospitalization: a systematic review. Ann Intern Med 2011;155:520-28.

18. Kansagara D, Englander H, Salanitro A, Kagen D, Theobald C, Freeman M, Kripalani S. Risk prediction models for hospital readmission: a systematic review. JAMA 2011;306(15):1688-98.

19. Zhou H, Della PR, Roberts P, Goh L, Dhaliwal SS. Utility of models to predict 28-day or 30-day unplanned hospital readmissions: an updated systematic review. BMJ Open 2016;6:e011060.

20. Department of Health. Long term conditions compendium of information. Department of Health, London; 2012.

21. Williams S, Bottle A, Aylin P. Length of hospital stay and subsequent emergency readmission. BMJ 2005; 331:371.

22. Van Walraven C, Dhalla IA, Bell C, Etchells E, Stiell IG, Zarnke K, Austin PC, Forster AJ. Derivation and validation of an index to predict early death or unplanned readmission after discharge from hospital to the community. CMAJ 2010;182(6):551-7.

23. Gruneir A, Dhalla IA, van Walraven C, Fisher HD, Camacho X, Rochon PA, Anderson GM. Unplanned readmissions after hospital discharge among patients identified as being at high risk for readmission using a validated predictive algorithm. Open Med 2013;5:e104-11.

24. Spiva L, Hand M, VanBracle L, McVay F. Validation of a predictive model to identify patients at high risk for hospital admission. J Healthc Qual 2016;38(1):34-31.

25. Wang H, Robinson RD, Johnson C, Zenarosa NR, Jayswal RD, Keithley J, Delaney KA. Using the LACE index to predict hospital readmissions in congestive heart failure patients. BMC Cardiovasc Disord 2014;14:97.

26. Cotter P, Bhalla VK, Wallis SJ, Biram RWS. Predicting readmissions: poor performance of the LACE index in an older UK population. Age Ageing 2012;41(6):784-9.

27. Kreilkamp, R. Application of the LACE risk assessment tool at Chinese hospital. The Advisory Board Group. http://www.avoidreadmissions.com/wwwroot/userfiles/documents/55/lace-risk-assessmenttool.pdf. Published 2011. Accessed 1st March 2014.

28. Tong L, Erdmann C, Daldalian M, Li J, Esposito T. Comparison of predictive modelling approaches for 30-day all-cause non-elective readmission risk. BMC Med Res Methodol 2016;16:26.

29. Yazaan-Ashoori P, Lee SK, Ibrahim Q, Van Spall HG. Utility of the LACE index at the bedside in predicting 30 day readmission or death in patients hospitalized with heart failure. Am Heart J 2016;179:51-8.

30. Bashir B, Schneider D, Naglak MC, Churilla TM, Adelsherger M. Evaluation of prediction strategy and care coordination for COPD readmissions. Hosp Pract 2016;44(3):123-8.

31. Mixon AS, Goggins K, Bell SP, Vasilevskis EE, Nwosu S, Schildcrout JS, Kripalani S. Preparedness for hospital discharge and prediction of readmission. J Hosp Med 2016;11(9):603-9.

32. Clarke A. Are readmissions avoidable? BMJ 1990;301:1136-8.

For peer review only

33. Drozda JP. Readmission rates. BMJ 2013;347.

34. Blunt I, Bardsley M, Dixon J. Trends in emergency admissions in England 2004-2009. Nuffield Trust, London: 2010.

35. Blunt I, Bardsley M, Grove A, Clarke A. Classifying emergency 30 day readmissions in England using routine hospital data 2004-2010: what is the scope for reduction? Emerg Med J 2014.

36. Billings J, Blunt I, Steventon A, Gheorghiou T, Lewis G, Bardsley M. Development of a predictive model to identify inpatients at risk of re-admission within 30 days of discharge (PARR-30). BMJ Open 2012; 00:e001667.

37. Van Walraven C, Wong J, Forster AJ. LACE+ index: extension of a validated index to predict early death or urgent readmission after hospital discharge using administrative data. Open Medicine 2012; 6(3):e90.

38. Donze JD, Williams MV, Robinson EJ, Zimlichman E, Avjesky D, Vasilevskis EE, Kripalani S, Metlay JP, Wallington T, Fletcher GS, Averbach AD, Schnipper JL. International validity of the HOSPITAL score to predict 30-day potentially avoidable readmission. JAMA Intern Med 2016;176(4):496-502.

39. Low LL, Liu N, Wang S, Thumboo J, Ong ME, Lee KH. Predicting 30 day readmission in an Asian population: building a predictive model by incorporating markers of hospitalization severity. PLoS One 2016;11(2):e0167413.

40. Garrison GM, Robelia PM, Pecina JL, Dawson NL. Comparing performance of 30-day readmission risk classifiers among hospitalized primary care patients. J Eval Clin Pract 2016;Epub ahead of print.

41. Szekendi MK, Williams MV, Carrier D, Hensley L, Thomas S, Cerese J. The characteristics of patients frequently admitted to academic medicine centers in the United States. J Hosp Med 2015;10(9):563-8.

For peer review only

STROBE 2007 (v4) Statement—Checklist of items that should be included in reports of *cohort studies*

Section/Topic	Item #	Recommendation	Reported on page #
**Title and abstract**	1	(*a*) Indicate the study's design with a commonly used term in the title or the abstract	1
		(*b*) Provide in the abstract an informative and balanced summary of what was done and what was found	2
**Introduction**			
Background/rationale	2	Explain the scientific background and rationale for the investigation being reported	4
Objectives	3	State specific objectives, including any prespecified hypotheses	4
**Methods**			
Study design	4	Present key elements of study design early in the paper	4-5
Setting	5	Describe the setting, locations, and relevant dates, including periods of recruitment, exposure, follow-up, and data collection	4-5
Participants	6	(*a*) Give the eligibility criteria, and the sources and methods of selection of participants. Describe methods of follow-up	4-5
		(*b*) For matched studies, give matching criteria and number of exposed and unexposed	N/A
Variables	7	Clearly define all outcomes, exposures, predictors, potential confounders, and effect modifiers. Give diagnostic criteria, if applicable	5-6
Data sources/ measurement	8*	For each variable of interest, give sources of data and details of methods of assessment (measurement). Describe comparability of assessment methods if there is more than one group	4-5
Bias	9	Describe any efforts to address potential sources of bias	N/A
Study size	10	Explain how the study size was arrived at	N/A: all patients in a two-year period were included
Quantitative variables	11	Explain how quantitative variables were handled in the analyses. If applicable, describe which groupings were chosen and why	6
Statistical methods	12	(*a*) Describe all statistical methods, including those used to control for confounding	6
		(*b*) Describe any methods used to examine subgroups and interactions	6
		(*c*) Explain how missing data were addressed	6
		(*d*) If applicable, explain how loss to follow-up was addressed	N/A

For peer review only

		(*e*) Describe any sensitivity analyses	N/A
**Results**			
Participants	13*	(a) Report numbers of individuals at each stage of study—eg numbers potentially eligible, examined for eligibility, confirmed eligible, included in the study, completing follow-up, and analysed	N/A: all patients in a two-year period were included
		(b) Give reasons for non-participation at each stage	N/A
		(c) Consider use of a flow diagram	N/A
Descriptive data	14*	(a) Give characteristics of study participants (eg demographic, clinical, social) and information on exposures and potential confounders	6
		(b) Indicate number of participants with missing data for each variable of interest	N/A: patient episodes with missing data were removed from the dataset
		(c) Summarise follow-up time (eg, average and total amount)	N/A
Outcome data	15*	Report numbers of outcome events or summary measures over time	6-7
Main results	16	(*a*) Give unadjusted estimates and, if applicable, confounder-adjusted estimates and their precision (eg, 95% confidence interval). Make clear which confounders were adjusted for and why they were included	6-9
		(*b*) Report category boundaries when continuous variables were categorized	6-9
		(*c*) If relevant, consider translating estimates of relative risk into absolute risk for a meaningful time period	N/A
Other analyses	17	Report other analyses done—eg analyses of subgroups and interactions, and sensitivity analyses	6-9
**Discussion**			
Key results	18	Summarise key results with reference to study objectives	9
**Limitations**			
Interpretation	20	Give a cautious overall interpretation of results considering objectives, limitations, multiplicity of analyses, results from similar studies, and other relevant evidence	10
Generalisability	21	Discuss the generalisability (external validity) of the study results	10-11
**Other information**			
Funding	22	Give the source of funding and the role of the funders for the present study and, if applicable, for the original study on which the present article is based	12

For peer review only

*Give information separately for cases and controls in case-control studies and, if applicable, for exposed and unexposed groups in cohort and cross-sectional studies.

Note: An Explanation and Elaboration article discusses each checklist item and gives methodological background and published examples of transparent reporting. The STROBE checklist is best used in conjunction with this article (freely available on the Web sites of PLoS Medicine at http://www.plosmedicine.org/, Annals of Internal Medicine at http://www.annals.org/, and Epidemiology at http://www.epidem.com/). Information on the STROBE Initiative is available at www.strobe-statement.org.

For peer review only
